# Sulfaphenazole reduces thermal and pressure injury severity through rapid restoration of tissue perfusion

**DOI:** 10.1038/s41598-022-16512-9

**Published:** 2022-07-23

**Authors:** Christopher T. Turner, Megan Pawluk, Juliana Bolsoni, Matthew R. Zeglinski, Yue Shen, Hongyan Zhao, Tatjana Ponomarev, Katlyn C. Richardson, Christopher R. West, Anthony Papp, David J. Granville

**Affiliations:** 1 International Collaboration on Repair Discoveries (ICORD) Centre, Blusson Spinal Cord Centre, Vancouver Coastal Health Research Institute, University of British Columbia, Rm 4470, 818 West 10th Ave., Vancouver, BC V5Z 1M9 Canada; 2 Department of Pathology and Laboratory Medicine, University of British Columbia, Vancouver, BC Canada; 3 Centre for Heart Lung Innovation, St. Paul’s Hospital, University of British Columbia, Vancouver, BC Canada; 4 Department of Cell and Physiological Sciences, University of British Columbia, Vancouver, BC Canada; 5 Department of Surgery, University of British Columbia, Vancouver, BC Canada; 6 British Columbia Professional Firefighters’ Burn and Wound Healing Laboratory, Vancouver Coastal Health Research Institute, Vancouver, BC Canada

**Keywords:** Inflammation, Translational research, Experimental models of disease, Geriatrics, Target identification, Antimicrobial responses, Inflammation, Blood flow

## Abstract

Pressure injuries, also known as pressure ulcers, are regions of localized damage to the skin and/or underlying tissue. Repeated rounds of ischemia–reperfusion (I/R) have a major causative role for tissue damage in pressure injury. Ischemia prevents oxygen/nutrient supply, and restoration of blood flow induces a burst of reactive oxygen species that damages blood vessels, surrounding tissues and can halt blood flow return. Minimizing the consequences of repeated I/R is expected to provide a protective effect against pressure injury. Sulfaphenazole (SP), an off patent sulfonamide antibiotic, is a potent CYP 2C6 and CYP 2C9 inhibitor, functioning to decrease post-ischemic vascular dysfunction and increase blood flow. The therapeutic effect of SP on pressure injury was therefore investigated in apolipoprotein E knockout mice, a model of aging susceptible to ischemic injury, which were subjected to repeated rounds of I/R-induced skin injury. SP reduced overall severity, improved wound closure and increased wound tensile strength compared to vehicle-treated controls. Saliently, SP restored tissue perfusion in and around the wound rapidly to pre-injury levels, decreased tissue hypoxia, and reduced both inflammation and fibrosis. SP also demonstrated bactericidal activity through enhanced M1 macrophage activity. The efficacy of SP in reducing thermal injury severity was also demonstrated. SP is therefore a potential therapeutic option for pressure injury and other ischemic skin injuries.

## Introduction

Pressure injuries, also known as bedsores or pressure ulcers, are areas of localized damage to the skin and underlying tissue due to shear force, pressure or friction. A hallmark of pressure injury pathogenesis is repeated cycles of ischemia–reperfusion (I/R). Ischemia is usually caused by bony prominences exerting pressure on surfaces such as wheelchair cushions for prolonged periods of time. By compressing the skin, blood flow is impeded thus preventing oxygen and nutrient supply. Blood flow is restored during reperfusion, triggering a burst of reactive oxygen species that damage blood vessels and surrounding tissues^1^. In severe cases blood flow is not restored, a phenomenon known as no-reflow.

I/R injury has been widely investigated in stroke, heart attack and spinal cord injury. These studies repeatedly demonstrate I/R to dramatically impact the health and recovery of affected tissues (reviewed in^2,3^). Meanwhile in pressure injury, I/R reportedly drives disease progression through increased inflammation, fibrosis and edema^4,5^. As such, therapeutics able to ameliorate the intensity and downstream effects of I/R are expected to greatly improve pressure injury outcomes.

Cytochrome P450 monooxygenases (CYPs), a family of hemeproteins involved in drug metabolism, generate superoxide radicals which cause cell damage and attenuate the bioavailability of nitric oxide (NO), a well-known vasodilator^6,7^. Reactive oxygen species produced by CYPs induce oxidative stress which causes various pathological conditions (reviewed in^8^). Studies investigating CYPs in cardiac I/R injury found cytochrome P450 monooxygenase 2C (CYP 2C) inhibitors to improve post-ischemic cardiac function and coronary flow, leading to reduced infarct size^9^. CYP 2C inhibition also reduced tissue injury by decreasing post-ischemic vascular dysfunction, reducing superoxide generation, increasing NO bioavailability and increasing blood flow^6^. One potent CYP 2C6 and CYP 2C9 inhibitor, sulfaphenazole (SP), an off patent sulfonamide antibiotic, reduced infarct size in a rat model of cardiac I/R injury^10^. SP is a potent inhibitor of human CYP 2C9 and CYP 2C6, a potential equivalent in rodents^11^. SP also restored endothelium-dependent vasodilation in diabetic mice^6^ and attenuated post-ischemic vascular dysfunction and peri-transplant ischemic injury in a murine model of cardiac allograft vasculopathy^7^.

Based on the dual-protective effect of this antibiotic on cardiac I/R injury, SP was predicted to have therapeutic applicability in pressure injury. In the present study, SP was evaluated in a model of I/R-mediated pressure injury. Apolipoprotein E knockout (ApoE−/−) mice are a mouse strain widely used in aging studies and associated with worsened ischemic outcomes^12,13^. ApoE−/− mice are also an accepted model of atherosclerosis, which increases with age and is associated with an elevated likelihood of acquiring a pressure injury in a hospital setting^14^. Thus, the utilization of magnet-induced I/R in ApoE−/− mice provided a useful model to investigate the therapeutic potential of SP in pressure injury, displaying many features representative of human pressure injury in an elderly population, including hyperproliferative epidermis, enhanced inflammatory cell infiltrate and decreased tissue perfusion.

## Results

### SP reduces thermal injury severity in ApoE−/− mice

**Figure 1:**
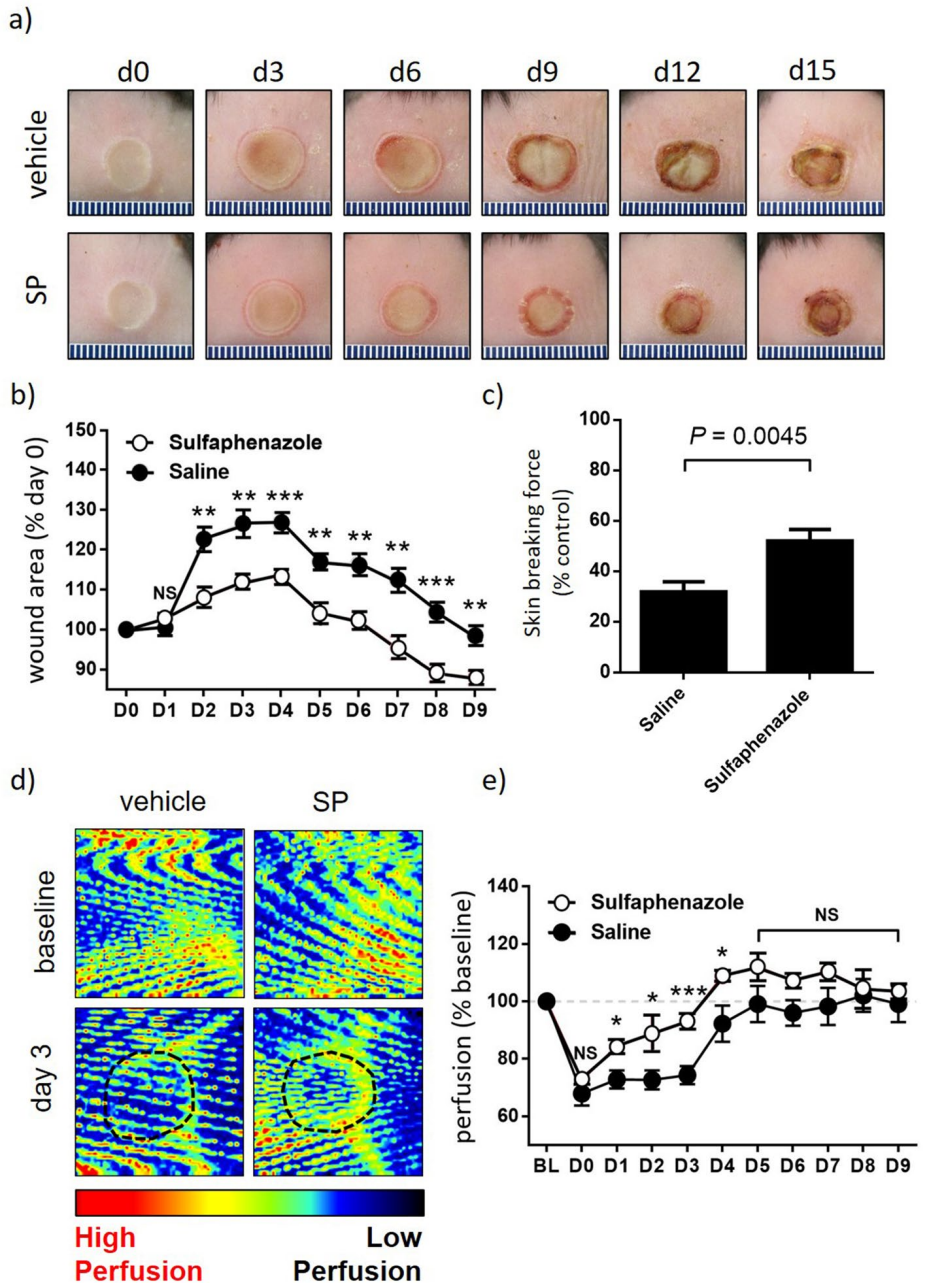
SP reduces burn injury severity. (**a**) Representative thermal injury photos. (**b**) Macroscopic measure of wound area with data presented as mean ± SEM at each time point, n = 6. (**c**) Minimum wound breaking force at d15 post-injury, n = 6 wounds per group. (**d**) Representative Doppler images of burn injury tissue. Region of interest (ROI) indicated by black dotted lines. (**e**) Quantification of tissue perfusion in ROI. Data evaluated as perfusion per unit area and presented as % baseline, mean ± SEM, n = 6. *P < 0.05, **P < 0.005, ***P<0.0001. Data analyzed by two-way ANOVA with Bonferonni post-hoc test.

To provide proof-of-concept for SP as an approach to improve tissue perfusion and improve wound healing in skin, SP was first evaluated in a mouse model of thermal injury. Wild-type C57Bl/6 mice were subjected to a grade 2 thermal injury as previously described^15^. SP was well tolerated with no adverse events noted. SP-treated mice displayed reduced wound area from day 2 to 9 post-injury compared to vehicle-treated controls (area under curve (auc) < 0.001, Fig. 1a,b), with this correlating with increased wound tensile breaking force at day 15 post-injury (*P* = 0.0045, Fig. 1c). Tissue perfusion was improved in the SP treatment group (auc < 0.001), with the most pronounced effect early in the wound healing cascade from day 1 to 4 post-injury (Fig. 1d,e).

### SP reduces pressure injury severity in ApoE−/− mice

Based on the effect of SP on tissue perfusion in burns, its therapeutic effect was next investigated in pressure injury. ApoE−/− mice, which have an enhanced susceptibility to ischemic injury^12,13^, were subjected to repeated rounds of I/R using a non-invasive technique involving skin compression with magnets as previously reported (Fig. S1a)^16^. Daily administration of SP via intraperitoneal injection had no apparent effect on overall mouse health. Mice lost < 10% body weight following 2 days of I/R which was maintained throughout the remainder of the study, with no difference observed between SP- and vehicle-treatment groups (Fig. S1b).

**Figure 2:**
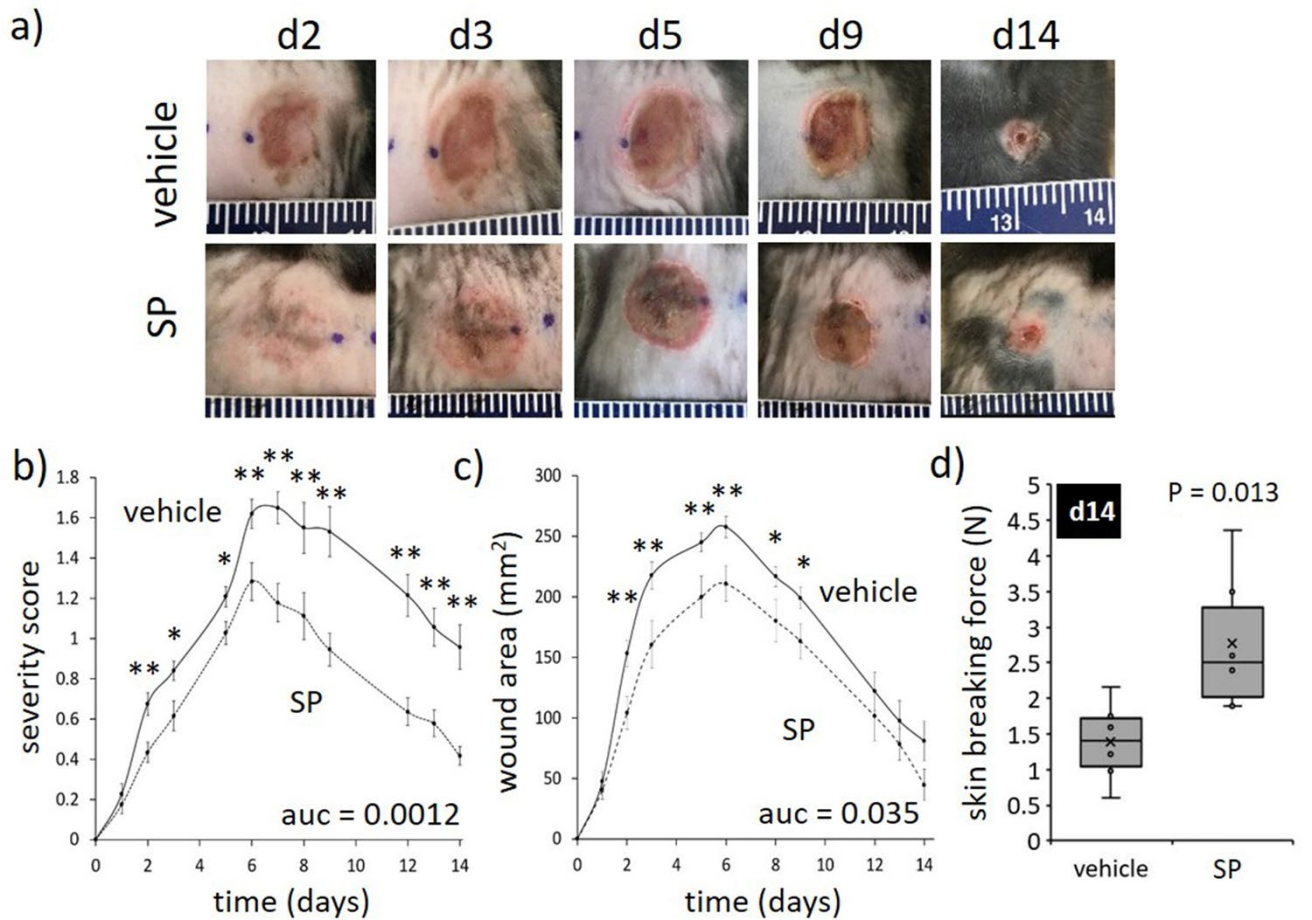
SP reduces I/R-mediated pressure injury severity. (**a**) Representative pressure injury photos. (**b**) Pressure injury severity score. (**c**) Macroscopic measure of wound area. Data in (**b**, **c**) displayed as SP-treated (dashed line) and vehicle-treated (solid line) and presented as mean ± SEM at each time point, n = 6. Auc = area under the curve. (**d**) Minimum wound breaking force at d14 post-injury, n = 6 wounds per group. Data presented as box and whisker plot. *P < 0.05, **P < 0.005. Data analyzed by two-way ANOVA with Bonferonni post-hoc test.

**Figure 3:**
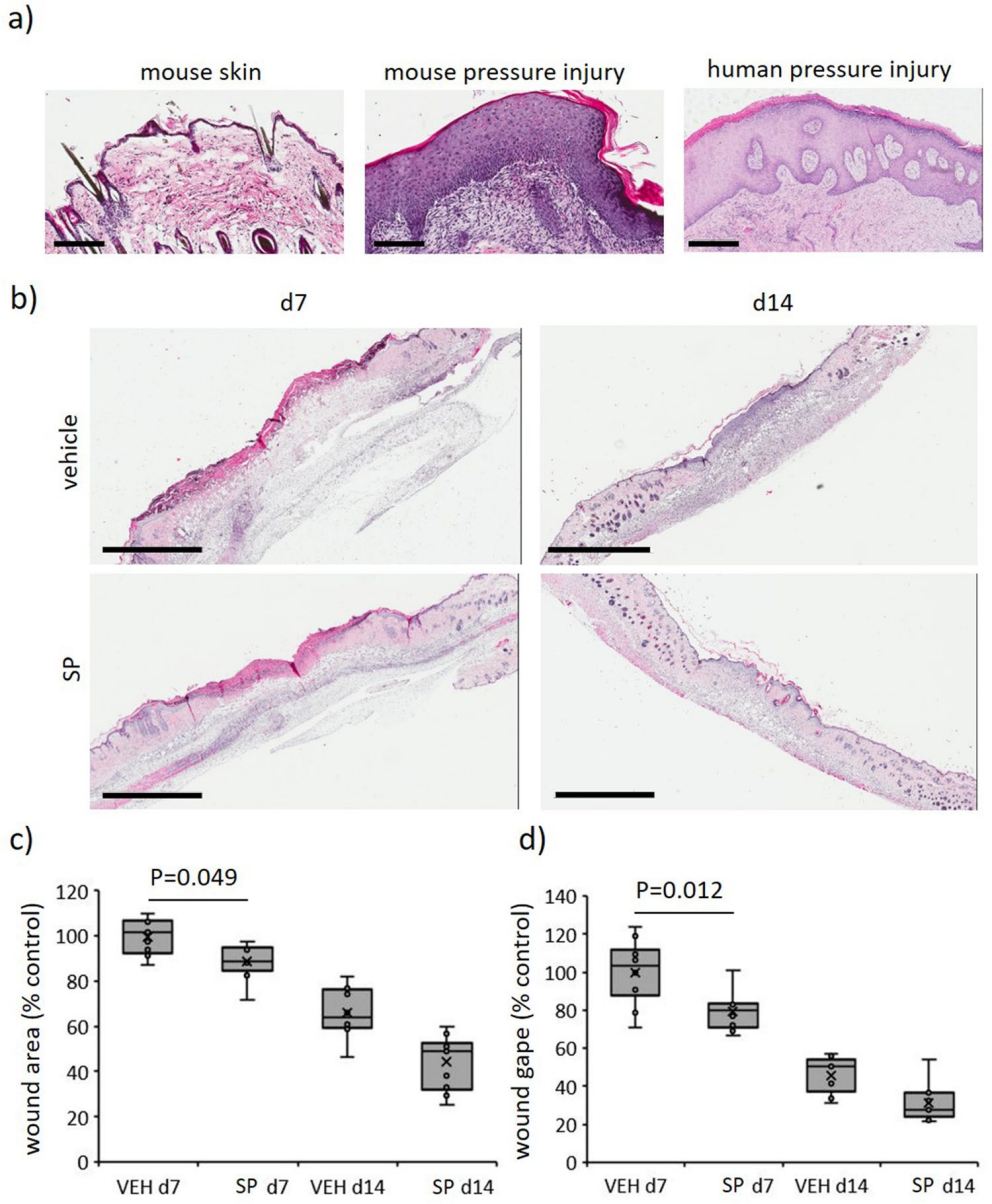
SP improves wound healing in I/R-mediated pressure injury. (**a**) Representative H&E images of mouse and human pressure injury tissue. Scale bars = 200 µm (mouse pressure injury and control at d14) and 500 µm (human pressure injury, stage 3, 66-year-old). (**b**) Representative H&E images of SP-treated mouse pressure injuries at d7 and d14. Scale bars = 2 mm. Wound area (**c**) and gape (**d**) quantified from H&E stained tissue. Presented as box and whisker plot, n = 6. Data analyzed by two-way ANOVA with Bonferonni post-hoc test.

A wound severity grading scale, providing a measure of injury depth, erythema and skin loss, was used to score mouse pressure injury (Table S1) as reported previously^17^. Vehicle-treated mice displayed increased wound severity (up to d8 post-injury initiation) and wound area (up to d6), approaching stage 2 before declining thereafter (Fig. 2a–c). SP-treated mice wounds displayed a similar pattern of increase as vehicle-treated mice, but both severity (auc = 0.0012, Fig. 2a,b) and area (auc = 0.035, Fig. 2a,c) were reduced. Skin breaking force at d14 post-induction of I/R was improved in SP- compared to vehicle-treated wounds (*P* = 0.013, Fig. 2d). Histologically, ApoE−/− mice pressure injury had increased inflammatory cell infiltrate and hyperproliferative epidermis (Fig. 3a), similar to human pressure injury. Both wound area (*P* = 0.049 at d7) and wound gape (*P* = 0.012 at d7) were reduced in SP-treated pressure injury at d7 post-initiation of I/R compared to vehicle-treated controls (Fig. 3b–d).

### SP increased tissue perfusion and reduced hypoxia in ApoE−/− mouse pressure injury

**Figure 4:**
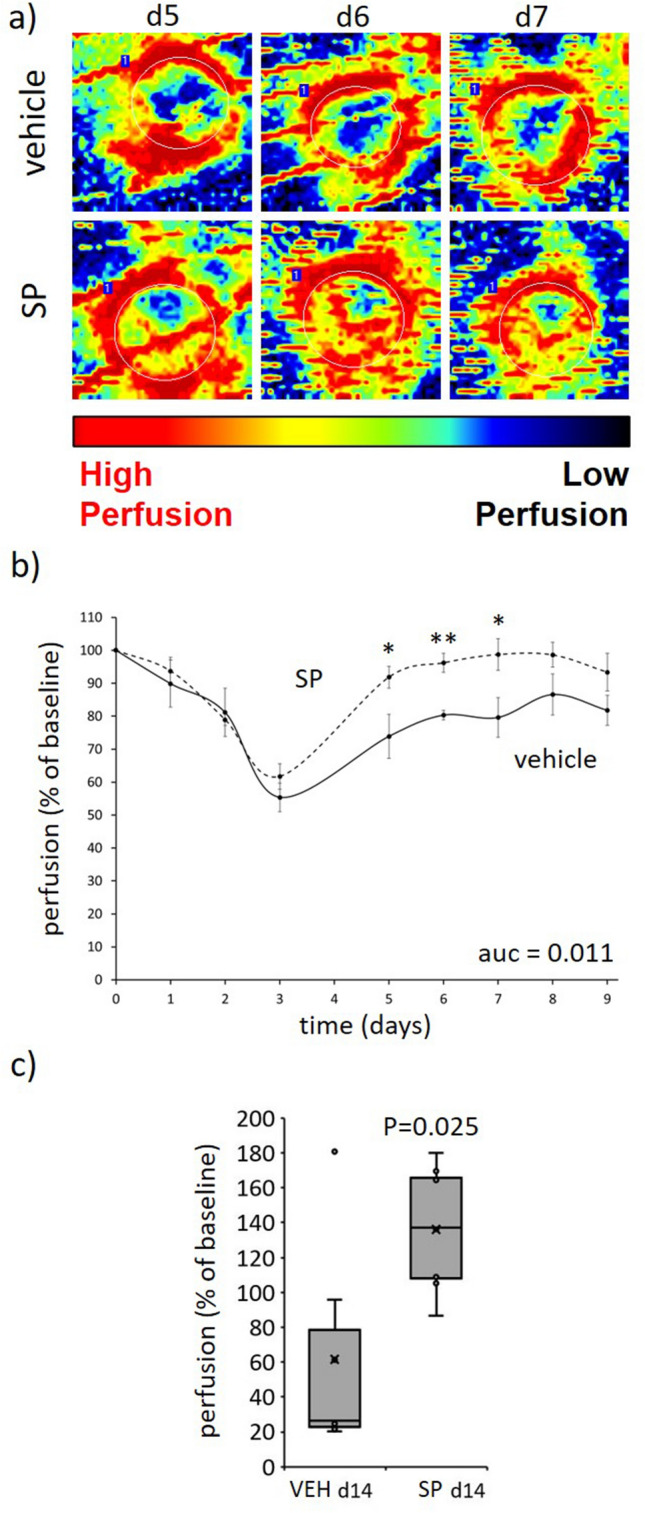
SP improves tissue perfusion post-injury. (**a**) Representative Doppler images of SP-treated pressure injury tissue. Region of interest (ROI) indicated by white dotted lines. (**b**, **c**) Quantification of tissue perfusion in ROI. Data presented as perfusion per unit area, mean ± SEM (**b**) or as box and whisker plot at d14 (**c**), n = 6. *P < 0.05, **P < 0.005. Data analyzed by two-way ANOVA with Bonferonni post-hoc test. Auc = area under the curve.

Over the three days required to induce I/R-mediated skin injury, tissue perfusion in both SP- and vehicle-treated mice was similarly reduced as measured by Doppler, suggesting equivalent depth of injury (Fig. 4a,b). From d3 onwards (i.e., post-I/R), SP-treated mice pressure injury displayed improved tissue perfusion at the wound margin and within the wound compared to vehicle-treated mice (auc = 0.011). Perfusion was unable to be determined from d10 to d13 as the Doppler was unable to penetrate the scabs that developed over the wounds, but by d14 when the scabs were no longer present perfusion remained elevated in SP-treated mice (*P* = 0.025; Fig. 4c).

**Figure 5:**
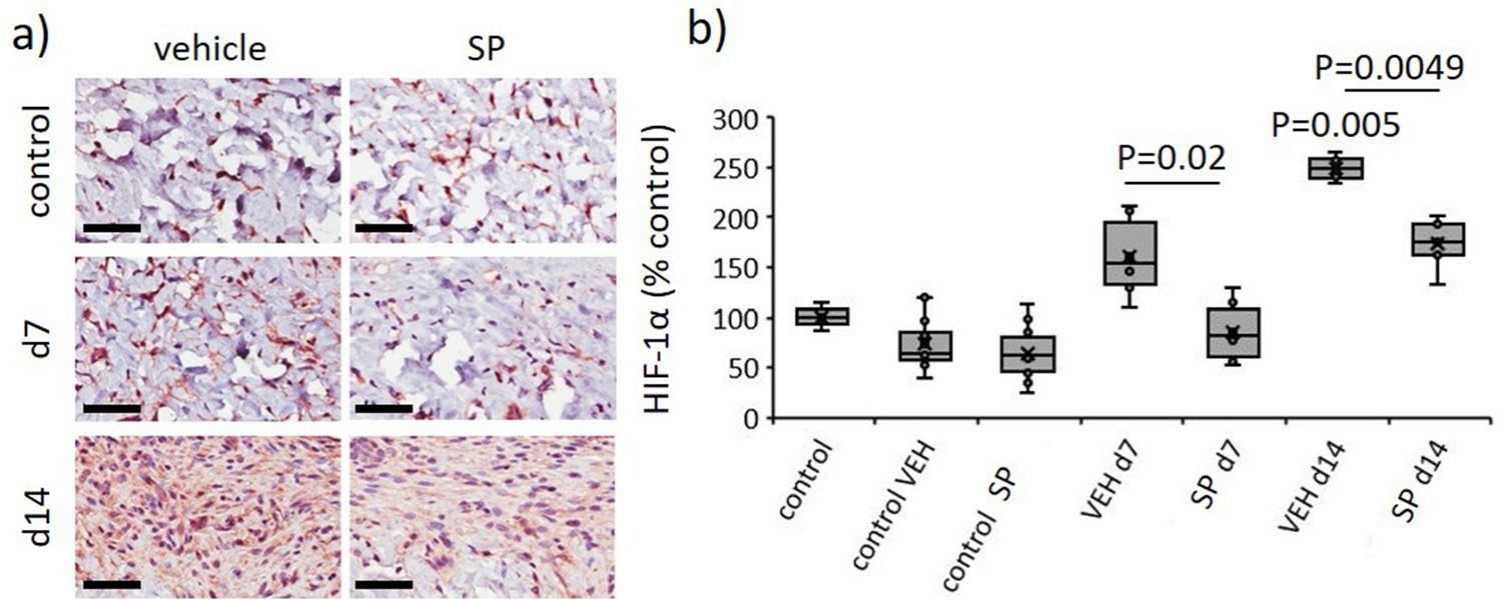
SP reduces hypoxia in response to I/R-mediated pressure injury. Representative images (**a**) and quantification (**b**) of HIF-1α in pressure injury wound tissue. Scale bar = 50 µm. HIF-1α data presented as percentage of vehicle-treated control, using box and whisker plot. Data were analyzed by two-way ANOVA with Bonferonni post-hoc test.

CYP 2C inhibition improves coronary blood flow and decreases post-ischemic vascular dysfunction^9^, suggesting SP may reduce hypoxia in I/R-mediated pressure injury. Hypoxia inducible factor-1 (HIF-1α) is rapidly degraded in normoxic conditions, but remains stable during periods of oxygen depletion, thus providing a direct marker of hypoxia. In vehicle-treated pressure injury, HIF-1α was elevated compared to unwounded skin (*P* < 0.005 at d14). Saliently, HIF-1α was reduced in SP- compared to vehicle-treated wounds (*P* ≤ 0.02 at d7 and d14, Fig. 5a,b).

### SP had no effect on endothelial cell apoptosis or microvascular permeability in ApoE−/− mouse pressure injury

As SP increased tissue perfusion and reduced hypoxia in pressure injury, SP may preserve the dermal vasculature through prevention of endothelial cell apoptosis. TUNEL+ cells were elevated in the dermis of mouse pressure injury at d7 and d14 post-initiation of injury compared to unwounded skin, however, there was no apparent difference between SP- and vehicle-treated pressure injury (Fig. S2).

**Figure 6:**
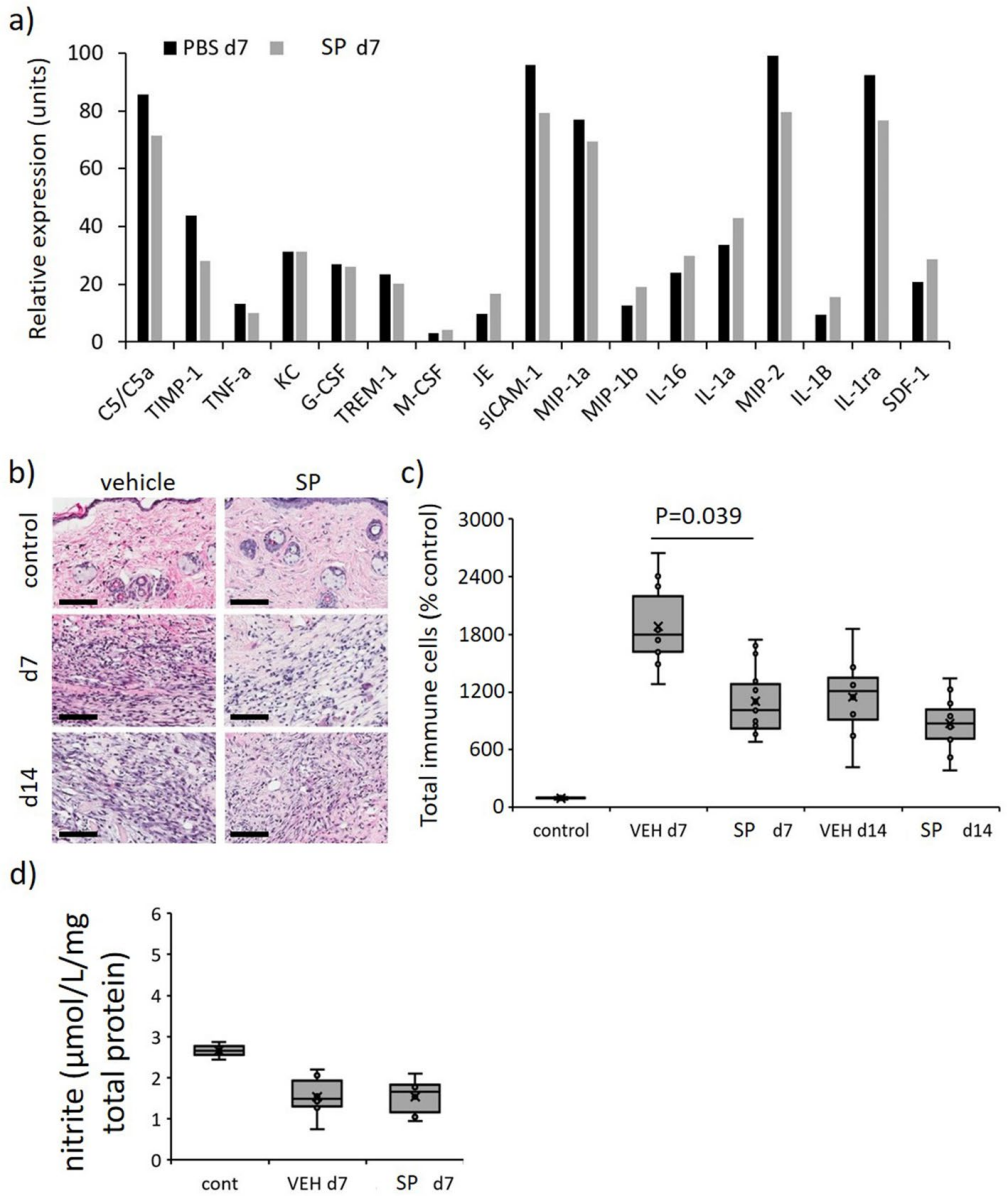
Reduced overall inflammation in SP-treated pressure injury. (**a**) Profiler screening of a panel of cytokines/chemokines in tissue extract from pressure injury at d7 post-initiation of I/R. Data displaying relative expression and expressed as mean pixel intensity, mean of n = 2 per time point per group. Total inflammatory cell infiltrate representative H&E stained tissue section images (**b**) and quantification (**c**) in mouse pressure injury. Scale bar = 100 µm. (**d**) Quantification of nitrite in pressure injury tissue extracts at d7. Data in (**c**, **d**) presented as a box and whisker plot and presented as total immune cells per unit area as a percentage of the unwounded vehicle-treated control (**c**) or µmol/L/mg total cell protein (**d**). Data were analyzed by two-way ANOVA with Bonferonni post-hoc test.

Hypoxia contributes to increased endothelial cell permeability^18^, thus the effect of SP on microvascular hemorrhage was examined. Soluble intercellular adhesion molecule-1 (sICAM-1), a biomarker of vascular wall inflammation^19^ and endothelial activation^20^, was abundantly expressed in vehicle-treated mice pressure injury at d7 identified by multiplex cytokine/chemokine screening (Fig. 6a). sICAM-1 levels were reduced in SP- compared to vehicle-treated pressure injury thereby indicating SP may indeed decrease I/R-mediated pressure injury endothelial cell permeability. However, Perl’s Prussian blue staining, which measures the deposition of interstitial hemosiderin, indicating increased microvascular permeability and micro-hemorrhage, displayed no difference between SP- and vehicle-treated mice (Fig. S3). Hypoxia also stimulates angiogenesis during wound repair (reviewed in^21^), thus CD31, a marker of angiogenesis, was investigated in pressure injury tissue of ApoE−/− mice. In both the wound area and wound edge, there was a small but non-significant reduction in angiogenesis in SP-treated at d7 (*P* = 0.28) and d14 (*P* = 0.38) compared to vehicle-treated mice (Fig. S4).

### SP reduced inflammation in ApoE−/− mouse pressure injury

Immune cell infiltration into the pressure injury wound area and surrounding margin was elevated in pressure injury compared to unwounded control skin (*P* < 0.005), with SP-treated wounds exhibiting an overall reduction compared to vehicle-treated mice pressure injury at day 7 (*P* = 0.039, Fig. 6b,c). Multiplex screening of inflammatory cytokines/chemokines in vehicle-treated pressure injury wound tissue extract at d7 also displayed increased expression of numerous pro-inflammatory markers of which complement component 5/5a (C5/5a), macrophage inflammatory protein (MIP)-1α, MIP-2 and interleukin-1ra (IL-1ra) were the most abundant (Fig. 6a**)**. Comparatively, the relative expression of each of these markers, in addition to tissue inhibitor of metalloproteinases-1 (TIMP-1), was reduced in SP-treated mice pressure injury.

Nitric oxide, a negative regulator of pro-inflammatory cytokine production and inflammatory cell recruitment (reviewed in^22^), may account for the reduced inflammation in SP-treated pressure injury. Notably, there was no difference in the detection of nitrite, an indirect measure of nitric oxide, in SP-treated compared to vehicle-treated pressure injury tissue extracts at d7 post-injury (Fig. 6d).

### SP increases activated macrophage recruitment and decreases bacterial load in ApoE−/− mouse pressure injury

**Figure 7:**
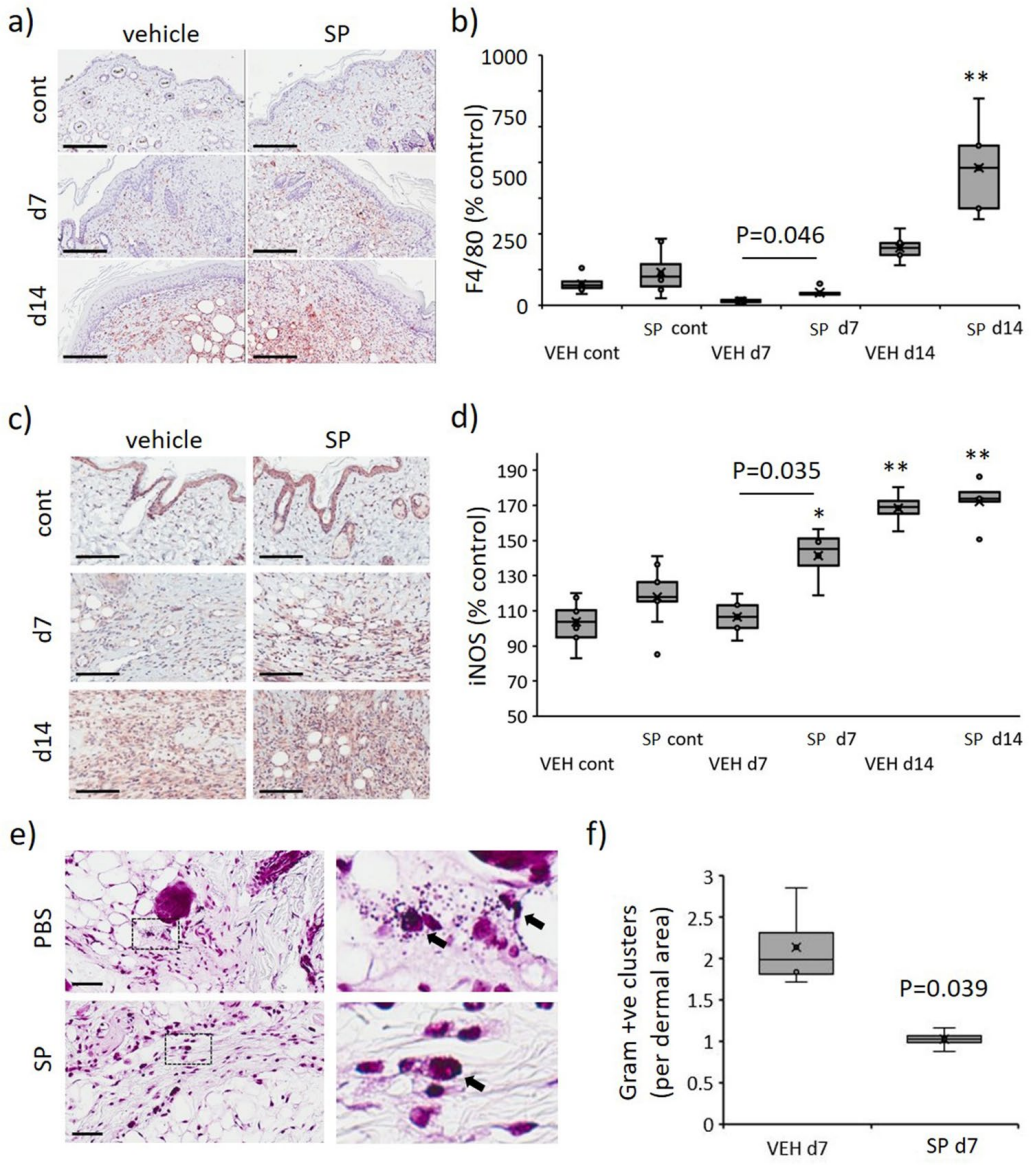
Increased macrophage recruitment and reduced bacterial load in SP-treated pressure injury. F4/80 representative images (**a**) and quantification (**b**) in mouse pressure injury. iNOS representative images (**c**) and quantification (**d**) in mouse pressure injury. Gram stain representative images, including high magnification panels (**e**), and quantification (**f**) in mouse pressure injury at d7 post-injury. Data in (**b**, **d**, **f**) presented as a box and whisker plot and analyzed by two-way ANOVA with Bonferonni post-hoc test (n = 6 per group). Data displayed as total staining intensity per unit area as a percentage of the unwounded vehicle-treated control (**b**, **d**) or number of bacterial colonies per unit area (**f**). * P < 0.05, ** P < 0.005. Scale bars = 200 µm (**a**), 80 µm (**c**) and 50 µm (**e**).

In contrast to the overall reduction in inflammation, SP-treated mice displayed increased F4/80 (macrophage marker) immune-positivity in the granulation tissue compared to vehicle-treated controls (*P* < 0.05 at d7, Fig. 7a,b). Assessment of pro-inflammatory markers at d7 post-injury indicated SP-treatment to increase several chemokines associated with macrophage recruitment, including JE (CCL2), MIP-1β (CCL4) and SDF-1 (CXCL12; Fig. 6a). Inducible nitric oxide synthase (iNOS), which is highly expressed by classically activated macrophages, was elevated in SP- compared to vehicle-treated pressure injury at d7 post-initiation of injury (*P* = 0.035), with no difference evident at d14 (Fig. 7c,d).

iNOS produced by activated macrophages is responsible for catalyzing the production of NO, which in turn mediates bactericidal actions^23^. Gram-stained pressure injury tissue sections displaying reduced bacterial colonies in SP- compared to vehicle-treated at d7 (*P* = 0.039, Fig. 7e,f).

### SP reduces pro-fibrotic activity in mouse pressure injury

**Figure 8:**
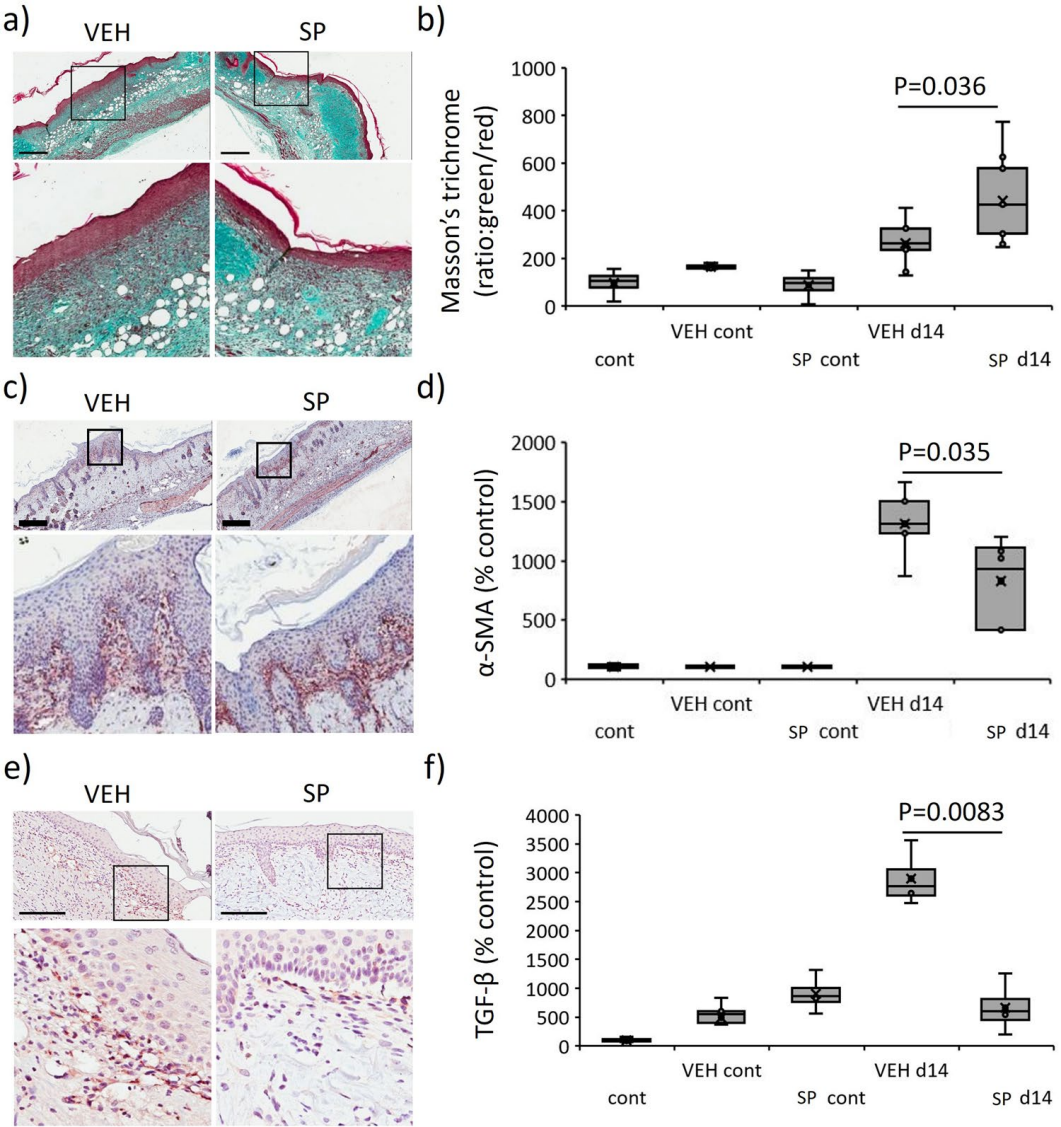
Reduced fibrotic activity in SP-treated pressure injury. Masson’s trichrome representative images (**a**) and quantification (**b**) in mouse pressure injury. α-SMA representative images (**c**) and quantification (**d**) in mouse pressure injury. TGF-β stain representative images (**e**) and quantification (**f**) in mouse pressure injury. Data in (**b**, **d**, **f**) presented as a box and whisker plot as ratio of green to red staining (**b**) or total staining intensity per unit area (**d**, **f**) as a percentage of the unwounded vehicle-treated control. Data were analyzed by two-way ANOVA with Bonferonni post-hoc test, (n = 6 per group). Scale bars = 200 µm.

Overexpression of HIF-1α induces fibrosis through increased myofibroblast differentiation, leading to excessive matrix synthesis and deposition^21^. As SP-treatment reduced hypoxia in mouse pressure injury, the role of SP in fibrosis was investigated. Masson’s trichrome of d14 pressure injury tissue displayed improved collagen maturation in SP- compared to vehicle-treated mice (*P* = 0.036, Fig. 8a,b). TIMP-1, a marker of fibrosis^24^, was reduced in SP- compared to vehicle-treated mouse pressure injury at d7 post-injury induction (Fig. 6a). α-SMA, a marker of myofibroblast recruitment, was reduced in SP- compared to vehicle-treated pressure injury (*P* = 0.035, Fig. 8c,d). TGF-β, an established driver of fibrosis, was elevated in vehicle-treated mouse pressure injury (*P* < 0.005) but this increase was absent in response to SP treatment (*P* = 0.0083, Fig. 8e,f).

## Discussion

In the current study, SP exposure post-injury was found to improve tissue perfusion in mouse models of both thermal injury and I/R-induced pressure injury, leading to reduced overall severity, improved wound closure and increased scar tensile strength. Daily administration was well tolerated in the mice, with no adverse outcomes identified. Together, SP may have therapeutic applications for pressure injury and other ischemic skin injuries.

SP did not prevent vasculature damage in response to I/R in ApoE−/− mice, rather SP rapidly increased tissue perfusion recovery back to pre-injury levels. This corresponded to reduced wound severity, improved overall appearance, decreased wound size, reduced inflammation and accelerated healing whilst also improving skin tensile strength. As I/R initiates the development of pressure injury and given the ability of SP to attenuate reperfusion injury, its safety profile and its bactericidal properties, SP could be a useful therapeutic for pressure injury treatment.

Mechanistically, SP increased tissue perfusion and reduced wound hypoxia. Oxygen is a key requirement for all stages of the wound healing cascade; thus, hypoxia is detrimental to tissue repair and regeneration. SP-mediated reductions in hypoxia were expected to directly contribute to the improved wound healing profile observed in the ApoE−/− mice. Although SP-mediated improvements in vascular perfusion have been reported in coronary blood flow^9^, to the best of our knowledge, this is the first time SP has been demonstrated to improve post-ischemic circulation in skin.

SP contributed to increased detection of iNOS in the pressure injury tissue. Elevated iNOS was expected to increase nitric oxide, which would enhance vasodilation, and thus provide a mechanistic explanation for the SP-mediated increase in tissue perfusion. However, we were unable to demonstrate elevated NO through the measure of nitrite. Detailed follow up studies are therefore required to confirm this outcome or identify alternative mechanisms of action.

SP reduced inflammatory cell recruitment during the pro-inflammatory phase of wound repair, whilst there was a concurrent decrease in numerous cytokines/chemokines involved in immune cell recruitment. In contrast, macrophages, including classically activated, were elevated in the SP treatment group. This discrepancy may be a result of the involvement of resident macrophages, rather than active recruitment of monocytes and other immune cells. Further studies are required to confirm the source of these macrophages in pressure injury.

Classically activated macrophages provide an important bactericidal role during the inflammatory phase of the wound healing cascade, either by engulfing infiltrating bacteria or through production of macrophage-derived reactive oxygen species. In the current study, SP-treatment increased macrophage detection in combination with reduced accumulation of gram-positive bacteria, confirming a potential bactericidal role. Notably, SP was FDA approved in the 1970’s based on earlier work investigating the treatment of leprosy and bacterial infections, providing a precedent for this antibacterial outcome^25^. At that time, SP was described as acting as a competitive inhibitor of DHPS, a folate synthesis enzyme present in bacteria, where inhibition led to folate deprivation and death^26^. In the current study, an additional mechanism for the bactericidal role for SP in wound healing, related to macrophage recruitment and activation, was therefore identified.

Neutrophils are primary responders following ischemia–reperfusion injury and expected to exacerbate pressure injury severity through release of reactive oxygen species, proteases, and various cationic peptides (reviewed in^27^). Moreover, neutrophils stimulate anti-inflammatory macrophage polarization (reviewed in^28^). As such, further studies to explore the effect of SP on neutrophil recruitment and function, and subsequent downstream effects on no-reflow, endothelial barrier dysfunction, and diapedesis may provide further mechanistic insight.

Fibrosis is associated with sustained local tissue hypoxia, with HIF-1α directly implicated in TGF-β1-mediated fibrogenesis^29^. I/R-mediated cardiac fibrosis has been documented (reviewed in^3^). As fibrosis is evident in pressure injury^30^, the role of SP in alleviating fibrosis was investigated. In the current study, SP-treatment improved collagen maturation and reduced myofibroblast recruitment. HIF-1α overexpression contributes to fibrosis through increased myofibroblast differentiation^21^, with hypoxia inducing the release of pro-fibrotic growth factors such as TGF-β from classically activated macrophages^31^. Our data confirmed a TGF-β-mediated fibrotic response to I/R-mediated pressure injury, whilst SP-treatment mitigated TGF-β elevation. Notably, although macrophages were elevated in SP-treated pressure injury there was no difference in activated macrophages during the remodeling phase of wound repair (d14), suggesting SP-treatment may induce a more rapid switch from a M1 (pro-inflammatory) to M2 (pro-healing) macrophage phenotype in response to I/R-mediated injury.

In the U.S, the total cost for pressure injury treatment is estimated at $26.8 billion per year, whilst the number of pressure injury acquired in hospital continues to climb^32^. Prevention followed by early detection is ideally the first line of defense. However, for those that do develop pressure injuries, the identification of treatments targeting early-stage pressure injuries is critically important to prevent pressure injuries from progressing to severe and difficult to manage wounds. Our results suggest SP as a viable therapeutic for pressure injury treatment.

## Materials and methods

### Study approvals

Animal studies approved by Animal Experimentation Committee of the University of British Columbia (A17-0024 and A17-0318). All experiments were performed in accordance with all relevant institutional guidelines and regulations. All methods are also reported in accordance with ARRIVE guidelines. Human skin and pressure injury obtained from Vancouver General Hospital Burns Clinic or Vancouver Coastal Health with approval from the University of British Columbia Human Research Ethics Committee (H12-00540) and after obtaining written, informed patient consent.

### Animals

C57Bl/6 mice and ApoE−/− mice (C57BL/6 background) were purchased from The Jackson Laboratory (Bar Harbor, ME, USA) and bred in-house. ApoE−/− mice were fed ad libitum a regular chow diet (equal parts PicoLab Mouse Diet 20: 5058 (9% fat) and PicoLab Rodent Diet 20: 5053 (5% fat), LabDiet, Richmond, IN, USA).

### Murine model of thermal injury

Grade 2 thermal injury was induced in female 8–10-week-old mice as previously described (n = 8 per treatment group)^15^. Briefly, mice were anaesthetized with inhaled isoflurane, the dorsum shaved, and hair removed with Nair. A 25 g, 1 cm in diameter metal rod, pre-heated in boiling water for 10 min, was lowered onto the skin and maintained under its own weight for 5 s. Wound photographs were captured on day 0, 3, 6, 9, 12 and 15. Animals were euthanized with inhaled isoflurane/CO_2_ asphyxiation at d15. Collected burn tissue was bisected with one half fixed in 10% (*v/v*) buffered formalin and processed so that the midpoint of the wound was sectioned. The other half was micro-dissected to remove unwounded skin then snap frozen in liquid nitrogen for protein extraction.

### Murine model of I/R-mediated pressure injury

Male, 15-week-old C57Bl/6 mice (n = 8 per treatment group) were anaesthetized with inhaled isoflurane, the dorsum was shaved then hair removed with Nair. A template was drawn on the dorsum to mark the location of the magnets assuring consistent placement for each animal. Skin was gently pinched between two identical round magnets (12 mm diameter, 5.0 mm thick, 1000 G magnetic force, 50 mm Hg compressive pressure; Master Magnetics, Castle Rock, CO, USA) to produce a 5 mm skin bridge. I/R cycles involved a 3 h period of magnet placement followed by a rest period of 30 min or overnight (Fig. S1a). Animals were not anesthetized or immobilized during I/R cycles and were allowed access to water and food ad libitum. Wound photographs were captured daily. Animals were euthanized with inhaled isoflurane/CO_2_ asphyxiation at d7 and d14. Collected pressure injury tissue was bisected with one half fixed in 10% (*v/v*) buffered formalin and processed so that the midpoint of the wound was sectioned. The other half was micro-dissected to remove unwounded skin then snap frozen in liquid nitrogen for protein extraction.

### SP-treatment of mice

Mice (n = 8 per treatment group) were administered SP once daily via intraperitoneal injection (100 µL). SP (5.13 mg/kg) was formulated immediately before use in PBS, pH 7.2, a dose based on its use in a rat model of cardiac ischemic injury^9^. An equivalent volume of PBS, pH 7.2, was administered as a vehicle-control. In the thermal injury trial, there was one experimental end-point (day 15), involving a total of 16 mice. In the pressure injury trial, there were two experimental end-points (day 7 and day 14), involving a total of 32 mice. In total, there were 48 mice included in this study. Sample size was based on an expected 20% improvement in wound healing (measured macroscopically from photographs and histologically from H&E stained wound sections), the major outcome measure. No animals were excluded from the study. Mice included in each mouse trial were randomly allocated to each treatment group, accounting for age, weight and litter. Experiments were performed at the same time of day, in the same surgical suite and using the same investigator team. All macroscopic analysis (and subsequent histological analysis) was performed by investigators blinded to the experimental groups.

### Reagents

Nair was from Church & Dwight (Ewing, NJ, USA). SP (4-Amino-N-(1-phenyl-1H-pyrazol-5-yl) benzenesulfonamide) was from Sigma (St. Louis, MO, USA). Protease inhibitor stock from Thermo Fisher Scientific (Waltham, MA, USA). Immunohistochemistry used antibodies as described (Table S2).

### Skin tensiometry

Mecmesin Motorised Force Tester (Mecmesin Corporation, Slinfold, UK) was used to evaluate tensile breaking force of burn and pressure injury tissue. Briefly, 1 × 4 cm of excised skin was loaded into a 200-N Spring Action Vice Clamp and separated at 3 cm/minute using the MultiTest 2.5-d Test System Stand. An Advanced Force Gauge 100 N assessed tensile strength in real time using Emperor Lite software. Tensile strength defined as the minimum force needed to trigger breakage of the skin.

### Morphometric analysis

Wound area measured in H&E stained slides as total wound area above the panniculus carnosus and below the epidermis, wound gape measured from one leading edge of the epithelium to the other. IHC intensity was calculated; staining intensity per unit area within two representative rectangles in wound area, as total positive cells per unit area within two representative 200 × 160 mm^2^ rectangles in wound section granulation tissues. Data presented as percentage of unwounded control samples. Inflammatory infiltrates assessed by ratio of blue (nuclear) to red (cytoplasmic) staining^33^. Vascular micro-hemorrhage was assessed as total hemosiderin positivity per unit area within the whole pressure injury tissue section. Mature collagen was determined in Masson’s trichrome stained slides as the ratio of green to red staining within two representative rectangles in the granulation tissue of wound sections, as above. Color deconvolution was used to isolate the colors for quantification using ImageJ software (NIH, USA). All slides blinded prior to analysis, n ≥ 4 images per group.

### Laser Doppler imaging

Perfusion assessed using the Periscan^®^ PIM 3 Blood Perfusion Imager apparatus, displaying blood perfusion as a numerical PU (volts) and as a color-coded image ranging from bright red (high) to dark blue (low). The settings were laser beam power = 1 mV; wavelength = 670 nm; resolution = 0.36 mm; scanner head distance = 5 cm; scanning area = 1.4 cm^2^; scanning time = 30 s.

### Immunohistochemistry and tissue staining

Immunohistochemistry was performed as previously reported^34^. Masson’s trichrome and Prussian blue staining were performed as reported previously^35^. TUNEL was performed as per the kit instructions. Images were captured using Aperio CS2 slide scanner (Leica, Wetzlar, Germany) and analyzed using Aperio ImageScope software (Leica Biosystems Inc, Lincolnshire, IL, USA).

### Nitrite assay

Tissue extracts prepared into PBS, pH 7.2, containing 1/100 dilution of protease inhibitor stock. Protein was removed from the samples by centrifugation using Amicon Ultra 0.5 mL spin columns (10,000 MW cut off, UFC 501024, Burlington, MA, USA) for 30 min at 4 °C. Nitrite was quantified in neat samples using Parameter Total Nitric Oxide and Nitrate/Nitrite Assay (R&D Systems, Minneapolis, MN, USA) as per the kit instructions.

### Proteome profiler

As per the kit instructions, proteome profiler antibody arrays (mouse cytokine array panel A, R&D Systems, Minneapolis, MN, USA) were applied to mouse tissue extracts (200 µg total cell protein), allowing detection of 44 inflammatory cytokines/chemokines. Only the most abundant (above the limit of detection) cytokines/chemokines were included in the analysis. Pressure injury tissue extracts from d7 were tested in duplicate and presented as the mean.

### Statistical analysis

Statistical differences in all experiments were determined using the ANOVA or Student’s t-test (2-sided, non-paired). Bonferroni post-test was used for group comparison analyses. *P* < 0.05 considered significant. For any data without a normal distribution, the Mann–Whitney U-test was performed. Error bars indicate the mean and SEM.

## Supplementary Information

Supplementary Information.

## Data Availability

Available from the corresponding author on reasonable request.
