# Supplementary material for: Sulfaphenazole reduces thermal and pressure injury severity through rapid restoration of tissue perfusion
Source: Sci Rep. 2022 Jul 23;12:12622. doi: 10.1038/s41598-022-16512-9 (PMC9308818; doi:10.1038/s41598-022-16512-9)
Supplement: Supplementary file 1 — Supplementary Information. [file 41598_2022_16512_MOESM1_ESM.docx]

**Table S1**. Pressure injury severity score in mice

| **Pressure injury severity score** | **Parameter** |
| --- | --- |
| 0 | Intact, healthy skin with normal capillary refill |
| 1 | Intact skin, non-blanchable erythema |
| 2 | Superficial/partial skin loss involving epidermis and dermis |
| 3 | Full thickness loss, damage and necrosis of subcutaneous tissue |
| 4 | Full thickness loss, extensive tissue necrosis, bone/muscle exposure |

**Table S2**. Antibodies for immunohistochemistry.

| **Antibody** | **Species** | **Final Concentration** | **Source and Catalog #** |
| --- | --- | --- | --- |
| HIF-1α | Mouse | 1/500 | Millipore Sigma, 07-628 |
| F4/80 | Mouse | 1/200 | Cell Signaling, Danvers, MA, USA |
| iNOS | Mouse | 1/1000 | Abcam, ab15323 |
| α-SMA | Mouse | 1/1000 | Abcam, ab5694 |
| TGF-β | Mouse | 1/50 | BD, 555052 |


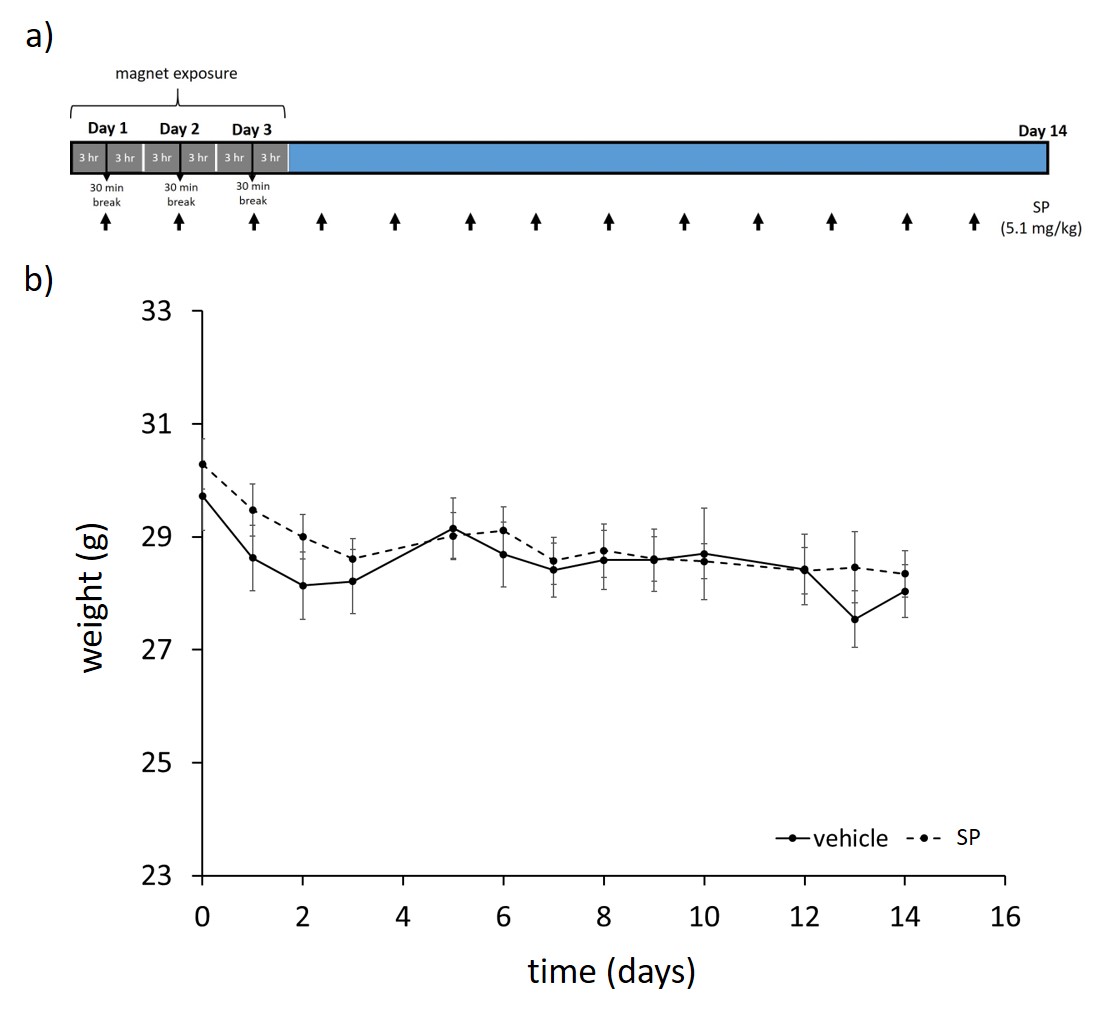


**Figure S1: Induction of I/R-Mediated Pressure Injury in ApoE-/- Mice.** (a) Flow chart of I/R-mediated pressure injury using the repeated application of magnets to skin. (b) Weight of SP- and vehicle-treated ApoE-/- mice, n=6 samples per group. Presented as weight (grams), mean ± SEM.

**
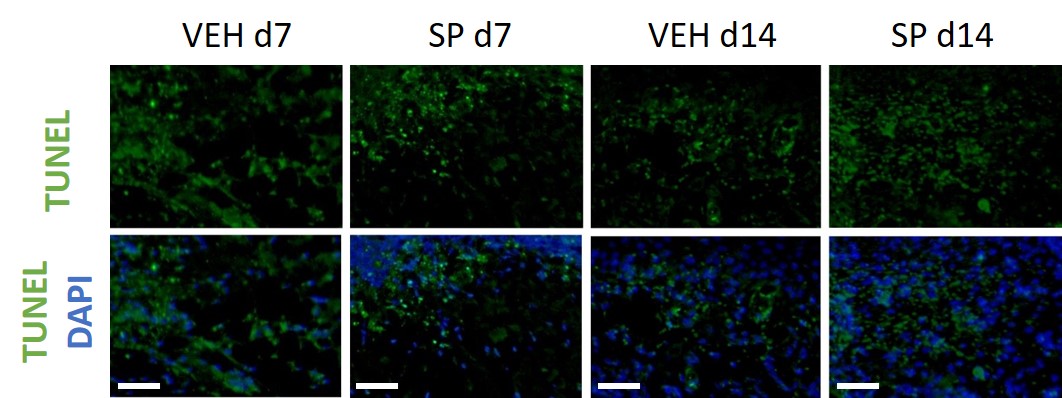
**

**Figure S2: SP Does Not Induce Cytotoxicity in ApoE-/- Mouse Pressure Injury.** Representative images of TUNEL in pressure injury wound tissue. Green is TUNEL+ staining, blue is DAPI nuclear stain. Scale bar = 50 µm.


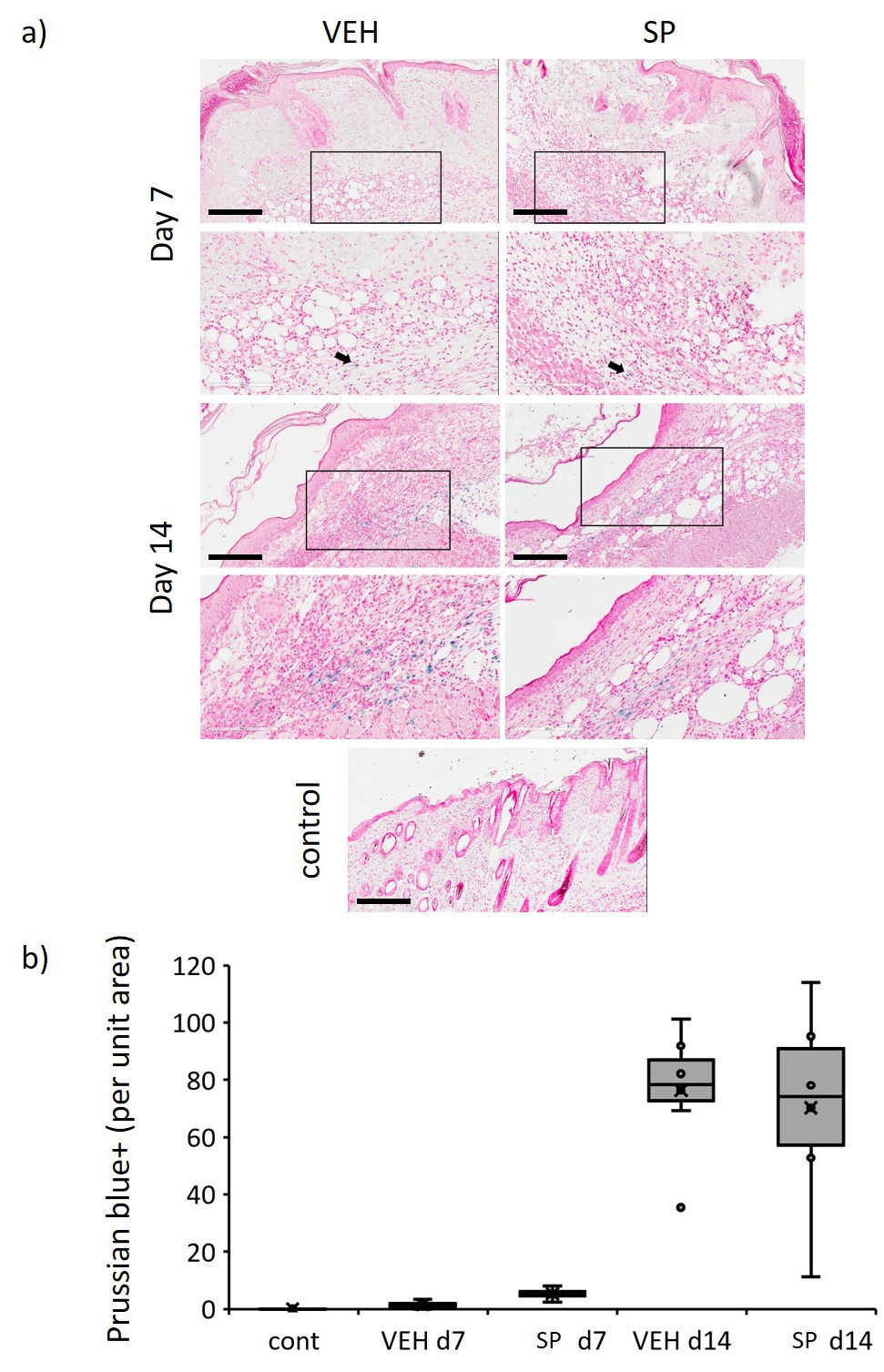


**Figure S3: SP Has No Effect on Micro-Hemorrhage in ApoE-/- Mice Pressure Injury.** (a) Representative images of Prussian blue staining. Scale bars = 300 µm. (b) Quantification of Prussian blue. Data analyzed by two-way ANOVA with Bonferonni post-test and presented in box and whisker plot as staining intensity per unit area in the dermis as a percentage of ApoE-/- unwounded control skin, n=6 per group.


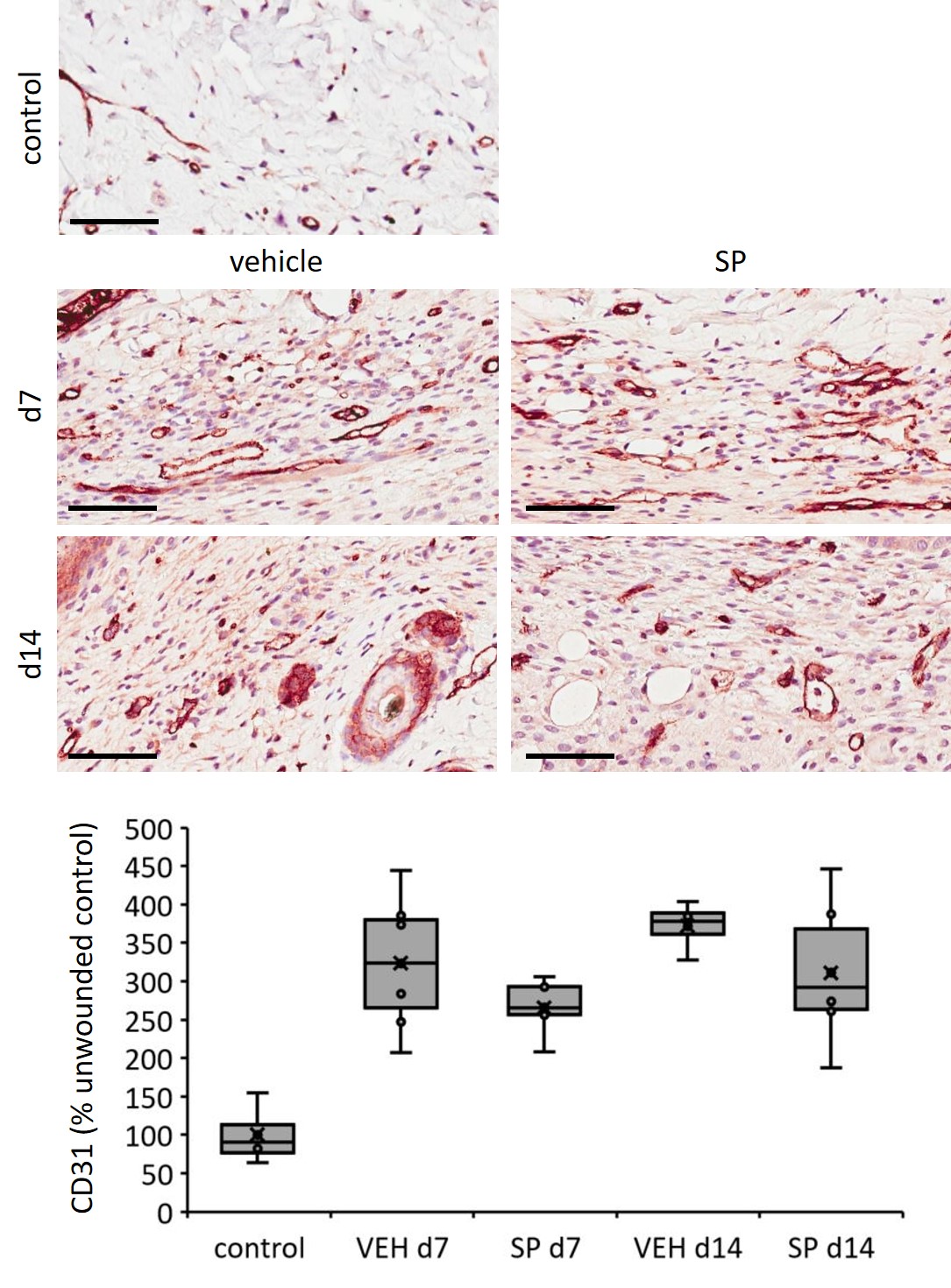


**Figure S4: SP Has No Effect on Angiogenesis in ApoE-/- Mice Pressure Injury.** (a) Representative images of CD31 immunohistochemistry in the wound area of ApoE-/- mice pressure injury ± SP and unwounded control skin. Scale bars = 60 µm. (b) Quantification of CD31. Data analyzed by two-way ANOVA with Bonferonni post-test and presented in box and whisker plot as staining intensity per unit area in the dermis as a percentage of ApoE-/- unwounded control skin, n=6 per group.
